# BDCA1+CD14+ Immunosuppressive Cells in Cancer, a Potential Target?

**DOI:** 10.3390/vaccines6030065

**Published:** 2018-09-19

**Authors:** Thomas J. van Ee, Heleen H. Van Acker, Tom G. van Oorschot, Viggo F. Van Tendeloo, Evelien L. Smits, Ghaith Bakdash, Gerty Schreibelt, I. Jolanda M. de Vries

**Affiliations:** 1 Department of Tumor Immunology, Radboud Institute for Molecular Life Sciences, Radboudumc, Nijmegen 6525 GA, The Netherlands; Thomas.vanEe@radboudumc.nl (T.J.v.E.); Tom.vanOorschot@radboudumc.nl (T.G.v.O.); gerty.schreibelt@radboudumc.nl (G.S.); 2 Laboratory of Experimental Hematology, University of Antwerp, Antwerp 2000, Belgium; Heleen.VanAcker@uantwerpen.be (H.H.V.A.); viggovantendeloo@gmail.com (V.F.V.T.); Evelien.Smits@uza.be (E.L.S.); 3 Center for Oncological Research, University of Antwerp, Antwerp 2000, Belgium; 4 Allergic Inflammation Discovery Performance Unit, Respiratory Therapy Area, GlaxoSmithKline, Stevenage SG1 2NY, UK; ghaith.x.bakdash@gsk.com; 5 Department of Medical Oncology; Radboud Institute for Molecular Life Sciences, Radboudumc, Nijmegen 6525 GA, The Netherlands

**Keywords:** BDCA1+CD14+ cells, cancer, dendritic cells, cancer immunotherapy, immune suppression, tumor microenvironment

## Abstract

Dendritic cell (DC) vaccines show promising effects in cancer immunotherapy. However, their efficacy is affected by a number of factors, including (1) the quality of the DC vaccine and (2) tumor immune evasion. The recently characterized BDCA1+CD14+ immunosuppressive cells combine both aspects; their presence in DC vaccines may directly hamper vaccine efficacy, whereas, in patients, BDCA1+CD14+ cells may suppress the induced immune response in an antigen-specific manner systemically and at the tumor site. We hypothesize that BDCA1+CD14+ cells are present in a broad spectrum of cancers and demand further investigation to reveal treatment opportunities and/or improvement for DC vaccines. In this review, we summarize the findings on BDCA1+CD14+ cells in solid cancers. In addition, we evaluate the presence of BDCA1+CD14+ cells in leukemic cancers. Preliminary results suggest that the presence of BDCA1+CD14+ cells correlates with clinical features of acute and chronic myeloid leukemia. Future research focusing on the differentiation from monocytes towards BDCA1+CD14+ cells could reveal more about their cell biology and clinical significance. Targeting these cells in cancer patients may improve the outcome of cancer immunotherapy.

## 1. Introduction

Dendritic cells (DCs) sample peripheral tissues for pathogens or malignant cells. Upon recognition of bacterial, viral, fungal, or tumoral danger signals, they process endocytosed samples for antigen-presentation. Whilst doing so, DCs migrate to the lymph node and mature towards strong activators of a T-cell response. Because of their capacity to activate naïve T-cells, DCs are commonly used in DC-based cancer immunotherapy [1]. These DC vaccines utilize the patient’s own DCs, which are ex vivo stimulated and loaded with tumor-specific antigens before being injected back into the patient, where they are expected to evoke a T-cell response against the tumor.

Although promising effects of DC vaccination are observed in clinical studies, the efficacy of DC vaccination remains limited by, amongst others, (1) the quality of the DC vaccine and (2) the immunosuppressive microenvironment of the tumor [2,3,4]. The latter consists of both cellular and soluble factors, such as myeloid derived suppressor cells (MDSCs), regulatory T-cells (T-regs) and interleukin (IL)-10 and expression of programmed cell death-1 ligands 1 and 2 (PD-L1 and PD-L2) [5]. In 2016, we identified a population of BDCA1+CD14+-immunosuppressive cells that was present both in blood and tumor of melanoma patients [6]. These cells are antigen-specific inhibitors of a tumor-targeted T-cell response and thus may affect DC vaccine efficacy. In this review, we summarize findings on BDCA1+CD14+ cells in solid tumors. In addition, we evaluate the presence of BDCA1+CD14+ cells in leukemic cancers. Because BDCA1+CD14+ cells were observed in melanoma and ovarian cancer, we hypothesize that BDCA1+CD14+ cells are present in a broad spectrum of cancers and that further research on their presence, generation, and biology may reveal new treatment opportunities and/or improvements for DC cancer immunotherapy.

## 2. Dendritic Cell Vaccination of Cancer

Dendritic cells are potent activators of an antigen-specific immune response by T-cells. DCs express receptors on their cell surface to recognize pathogens and foreign antigens. DCs sample peripheral tissues, where they take up the antigen, degrade it into smaller peptides by means of proteolysis, and load them onto major histocompatibility complex (MHC) class I or II molecules. In general, intracellular antigens are presented in MHC class I to CD8+ T-cells, whereas antigens of extracellular origin are presented in MHC class II to CD4+ T-cells. The binding of ‘danger signals’ to pathogen recognition receptors, like Toll-like receptors (TLRs), increases the efficiency of the antigen-processing and -presentation machinery and the expression of homing receptors needed for migration towards secondary lymphoid tissues i.e., lymph nodes. During migration, DCs mature; they lose their capacity to take up antigens, increase surface expression of MHC class I or II molecules and costimulatory molecules, increase secretion of proinflammatory cytokines, and more profoundly express their finger-like dendrites to increase their cell surface area. All of this increases their efficiency to interact with T-cells within the lymph node. 

Due to their capacity to evoke an antigen-specific T-cell response against tumor cells, DC vaccines are used in cancer immunotherapy [7]. DCs are ex vivo loaded with tumor-specific antigens and are matured under the influence of danger signals such as tumor necrosis factor-α, IL-1β and IL-6 or TLR ligands. This mimics the in vivo sampling of DCs for tumor-specific (neo-)antigens and their subsequent maturation [4]. After isolation, loading, and maturation, the DCs are injected back into the patient, where they can evoke an anti-tumor T-cell response in an antigen-specific manner [3].

The most commonly used method to prepare DC vaccines is in vitro differentiation of monocytes towards monocyte-derived DCs (moDCs) under the influence of IL-4 and granulocyte-macrophage colony-stimulating factor, followed by maturation and antigen loading [4]. However, this method demands an extensive differentiation period. Although numerous clinical studies proved the safety and immunogenicity of tumor-associated antigens introduced by DCs, limited clinical success has been achieved with moDC vaccines. Recently, the use of natural occurring circulating DCs (nDCs) has become feasible, due to the development of efficient isolation techniques [8]. nDCs offer potential advantages of both more standardized manufacturing, which enables application in multicenter trials, and increased immune potency. In peripheral blood, two major types of nDCs can be distinguished, plasmacytoid DCs (pDCs) and myeloid DCs (mDCs). mDCs can be further subdivided based on surface expression of BDCA1 and BDCA3 [9]. pDCs are specialized in dealing with viral infections, secrete large amounts of interferon-α/β and induce maturation of B cells towards antibody-producing plasma cells [8,10,11]. mDCs are specialized in immunity against bacteria and fungi and secrete large amounts of the T helper (Th)1-skewing cytokine IL-12 upon triggering of TLRs [12,13,14]. Combined, these DC subsets have shown to possess complementary functions [8,15,16,17,18]. Whereas, moDCs need to be differentiated ex vivo, nDCs can immediately be matured and loaded with antigens after isolation. Preparation of nDC vaccines is therefore less time-consuming and labor-intensive and the highly standardized isolation method facilitates multicenter application in the future. Our recent phase I studies in melanoma patients demonstrated that vaccination with pDCs or mDCs is feasible and safe, and it shows promising clinical results [11,19]. 

## 3. Tumor Immune-Evasion in Cancer

Cancer immunotherapy is a rapidly evolving field, but still not beneficial to all patients. One component that contributes to this rather disappointing outcome is tumor immune evasion. Tumors create a suppressive tumor microenvironment (TME), where immune cells and tumor cells are in a constant battle with each other [5]. This battle results in a selection of tumor cell variants that have gained the ability to evade the immune system by means of active immunosuppression [20,21]. A tumor that manages to keep the immune system at bay has a higher survivability and proliferative rate, while also being more prone to recurrence. Factors that contribute to tumor immune evasion include loss of tumor antigen expression or downregulation of MHC class I molecules, secretion of suppressive cytokines, and recruitment of suppressive immune cells, such as T-regs, type II macrophages, and MDSCs [22,23,24].

## 4. BDCA1+CD14+ Immunosuppressive Cells

Recently, we identified a novel population of immunosuppressive myeloid cells that co-expresses BDCA1 and CD14 in blood and tumors of cancer patients [6]. This cell population was first identified in BDCA1+ DC vaccines that were used to treat metastatic melanoma patients in a clinical trial performed by our group [19]. In addition to BDCA1+ myeloid DCs, the DC vaccines contained between 6% and 45% BDCA1+CD14+ cells [6]. Further analysis revealed that BDCA1+CD14+ cells were not only present in peripheral blood of stage III and stage IV melanoma patients, but that metastatic lesions in the skin, lymph nodes, and colon also showed increased frequencies of these cells. In addition, BDCA1+CD14+ cells were also found in inflammatory tumor ascites from ovarian cancer patients [6]. BDCA1+CD14+ cells are also present in blood of healthy donors, but at much lower levels than in cancer patients [6,25]. 

Interestingly, melanoma patients that were vaccinated with DC vaccines with a content of more than 25% BDCA1+CD14+ cells showed a reduced T-cell response to the immunomonitoring molecule Keyhole Limpet Hemocyanin (KLH) compared to patients whose DC vaccines contained less than 25% BDCA1+CD14+ cells. These data suggested that BDCA1+CD14+ cells may induce immune suppression and that the presence of these cells may thus hamper the induction of an effective anti-tumor immune response by the DC vaccine. In vitro experiments confirmed that BDCA1+CD14+ cells were indeed able to suppress proliferation of KLH-specific T-cells [6]. 

Their expression of CD14 suggests a joint origin with MDSCs, that are also significantly elevated in melanoma patients. However, whereas MDSCs suppress T-cell proliferation in a broad manner, in vitro studies showed that BDCA1+CD14+ cells suppress T-cell proliferation in an antigen-specific manner. Furthermore, BDCA1+CD14+ cells expressed high levels of the inhibitory immune checkpoint molecule PD-L1 and the MHC class II molecule HLA-DR, in contrast to MDSCs that have low HLA-DR expression [26]. 

Morphologically, BDCA1+CD14+ cells resemble BDCA1+ DCs. They display phenotypical similarities, including high expression levels of CD11c and HLA-DR [6,25]. However, in contrast to BDCA1+ DCs, BDCA1+CD14+ cells also express the monocytic marker CD11b, although at lower levels than monocytes. Transcriptome analysis revealed that BDCA1+CD14+ cells are closely related to both monocytes and BDCA1+ DCs, but constitute a distinct cell population [6]. Functionally, BDCA1+CD14+ have the ability to take up antigens, mature in response to TLR stimulation and activate and polarize naïve CD4+ T-cells into Th1 cells. The T-cell-stimulatory capacity of BDCA1+CD14+ cells is less compared to BDCA1+ mDCs [6,25]. This may at least partially be attributed to PD-L1 expression on BDCA1+CD14+ cells, as neutralizing PD-1 on these cells significantly enhanced BDCA1+CD14+ cell-induced T-cell proliferation [6]. 

The origin of BDCA1+CD14+ cells is unclear. In vitro experiments showed that monocytes can differentiate towards cells phenotypically resembling BDCA1+CD14+ cells when cultured in the presence of serum of melanoma patients. Interestingly, also BDCA1+ DCs cultured with serum of melanoma patients started co-expressing CD14 [6]. Thus, circulating BDCA1+CD14+ may arise from either monocytes or BDCA1+ DCs or they may even have a mixed origin. Immunosuppressive factors secreted by tumor cells could drive this differentiation. Candidate factors include tumor-secreted molecules previously implicated in MDCS formation, such as GM-CSF, VEGF, IL-1β, IL-6, and prostaglandin E2 [24,27], but other tumor-derived factors may be involved.

Transcriptome analysis revealed similarities between BDCA1+CD14+ cells and macrophages. However, macrophages are by definition tissue-resident cells, whereas BDCA1+CD14+ cells are found in blood as well. More resemblance was noticed with CD14+ dermal DCs (CD14+DDCs), which also circulate in the periphery and possess low immunostimulatory competence and induce immune tolerance [28,29,30,31]. CD14+ DDCs and circulating BDCA1+CD14+ cells may be related, since CD14+DDCs express BDCA1 and can be generated from monocytes by coculture with endothelial cells [32,33]. 

## 5. BDCA1+CD14+-Immunosuppressive Cells in Leukemic Cancers

Since tumor-associated factors induce the differentiation of monocytes or BDCA1+ DCs into BDCA1+CD14+ cells, we hypothesized that BDCA1+CD14+ cells are also present in other cancers, such as leukemia. Leukemia is characterized by the suppression of normal blood cell development due to the outnumbering of these cells by leukemic blast cells. This results in anemia, tiredness, immune disorders, and weight loss. 

**Figure 1:**
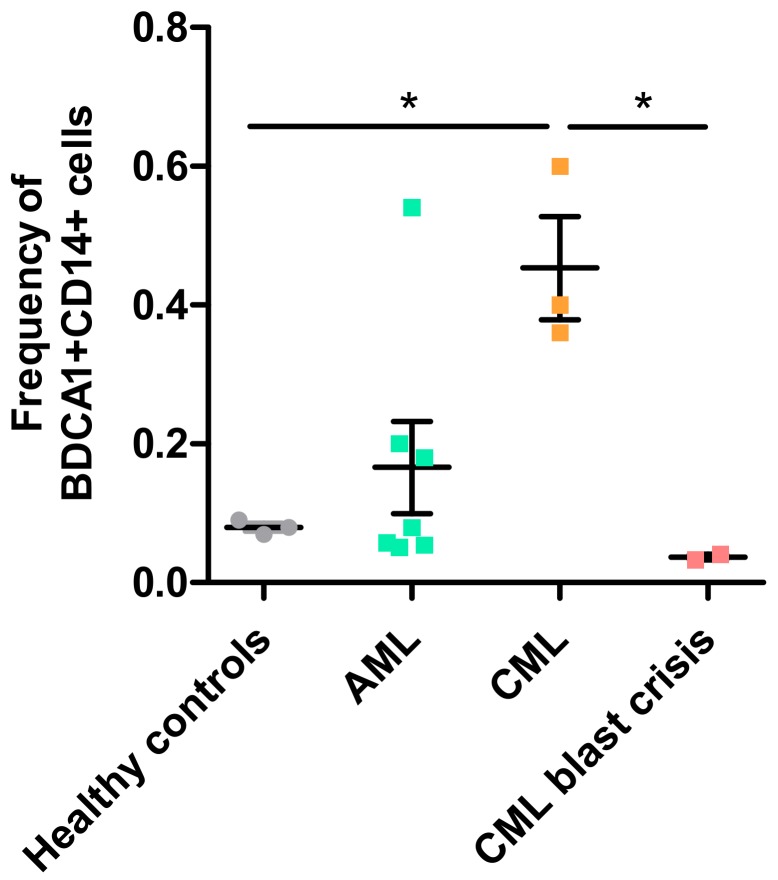
Frequency of BDCA1+CD14+ cells in peripheral blood mononuclear cells (PBMCs) of leukemia patients. Frequency of cells was analyzed by flow cytometry in PBMCs of healthy controls (*n* = 3) and patients with acute myeloid leukemia (AML; *n* = 7), chronic myeloid leukemia (CML; *n* = 3) or a CML blast crisis (*n* = 2). Each dot represents PBMCs of a single patient. Mean ± SD. * *p* < 0.05%; One-Way ANOVA with Tukey’s multiple comparison test).

Various subtypes of leukemia exist, which are mainly classified as being acute or chronic and of myeloid or lymphoid origin. In acute leukemia, there is a proliferation of undifferentiated immature hematopoietic cells (called blasts) in the bone marrow that lost the ability to differentiate into mature blood cells. In contrast, in chronic leukemia there is an increase in more mature myeloid or lymphoid cells in the bone marrow and blood that still have the capacity to fully differentiate into granulocytes or lymphocytes. Further discrimination between leukemia types depends on the origin and/or stage of the leukemic cells. We evaluated the presence of circulating BDCA1+CD14+ cells in patients with acute myeloid leukemia (AML), chronic myeloid leukemia (CML), acute lymphatic leukemia (ALL), chronic lymphatic leukemia (CLL), biphenotypical acute leukemia (B- and T-ALL), and idiopathic myelofibrosis (IMF, rise of CD34+ hematopoietic stem cells) (Figure 1; methods in Appendix A). Peripheral blood mononuclear cells (PBMCs) of CML patients showed increased frequencies of BDCA1+CD14+ cells when compared to PBMCs of healthy controls (*p* < 0.05; Figure 1). PBMCs of AML patients also showed a slight, but not significant, increase.

**Figure A1:**
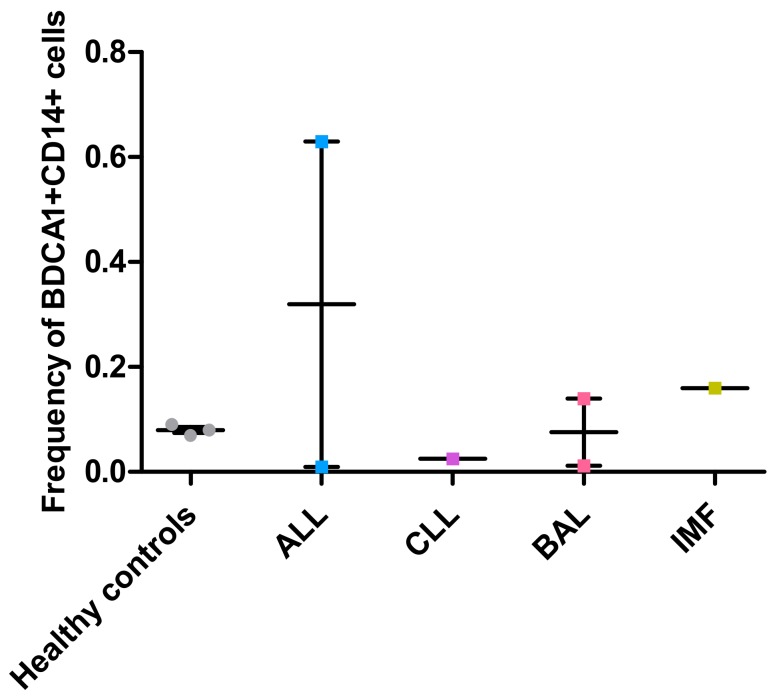
Frequency of BDCA1+CD14+ cells in PBMCs of leukemia patients. Frequency of cells was analyzed by flow cytometry in BMPC of healthy controls and patients with acute lymphoid leukemia (ALL; *n* = 2), chronic lymphoid leukemia (CLL; *n* = 1), biphenotypical leukemia (BAL; *n* = 2) or idiopathic myelofibrosis (IMF; *n* = 1). Each dot represents PBMCs of a single patient. Mean ± SD.

Preliminary results suggest that the presence of BDCA1+CD14+ cells correlates with clinical features of acute and chronic myeloid leukemia. CML patients endure less severe clinical symptoms than AML patients and in some cases do not present any clinical symptoms at all [34,35]. However, a CML patient can progress to a ‘blast crisis’, an increase of lymphoblastic cells, in which the suppression of normal blood cells is comparable to that of AML and the same clinical symptoms as in AML are observed. In line with the clinical symptoms, the presence of BDCA1+CD14+ was also reduced in patients with CML blast crisis when compared to CML (Figure 1). The frequencies of BDCA1+CD14+ cells in CML were higher than in AML but reduced to levels observed in AML during CML blast crisis. Possibly, the presence of BDCA1+CD14+ cells explains the persistent nature of CML; antigen-specific immunosuppression by BDCA1+CD14+ cells may hamper the clearance of leukemic cells by the immune system. This seems contradictory; a rise of leukemic cells could be considered as an increased tumor-size, which could result in an increased tumor-induced differentiation of monocytes towards BDCA1+CD14+ cells. However, the expansion of leukemic cells suppresses the formation of other functional cells, such as monocytes, and could thus limit the expansion of BDCA1+CD14+ cells. Due to low patient numbers, no conclusions could be drawn from results that were obtained with PBMCs from patients with ALL, CLL, BAL, or IMF (Figure A1). Future studies should reveal whether BDCA1+CD14+ cells are also present in these types of leukemia and if these cells have the same immunosuppressive capacity, as was shown in melanoma patients.

## 6. Conclusions and Future Perspectives

DC vaccination is applied in clinical studies in various cancer types, including melanoma, colon carcinoma, prostate cancer, mesothelioma, multiple myeloma, and leukemia [3,4,36,37,38,39]. The recently characterized BDCA1+CD14+ immunosuppressive cells may hamper the efficacy of DC-based immunotherapy in cancer patients, due to (1) their presence in cancer vaccines, especially cancer vaccines that include BDCA1+ DCs and (2) local and systemic suppression of tumor-specific immune responses. Anti-tumor responses induced by DC-based vaccines may therefore be improved by (1) removal of these cells from DC vaccines and (2) counteracting their suppressive effect in vivo. 

Removal of BDCA1+CD14+ cells from DC vaccines has already been implemented in our ongoing DC vaccination studies in melanoma and prostate cancer patients (clinicaltrials.gov identifiers NCT02993315, NCT02574377, NCT02692976). In these studies, patients are vaccinated with either pDCs, BDCA1+ mDCs or a combination of both natural DC subsets. In contrast to the first clinical trials with BDCA1+ mDCs in melanoma patients [19], in which the DC vaccines also contained BDCA1+CD14+ cells [6], we incorporated a CD14-depletion step in the DC vaccine manufacturing protocol in our ongoing studies to remove BDCA1+CD14+ cells from the vaccine. Thus, only immunostimulatory DCs are administered to the patients.

To increase the efficacy of immunotherapy, the local presence or suppressive action of BDCA1+CD14+ cells should also be reduced, either by inhibiting the differentiation of monocytes towards BDCA1+CD14+ cells or by targeting the BDCA1+CD14+ cells themselves. As we previously reported [6], monocytes differentiate towards BDCA1+CD14+ cells when culturing monocytes with serum of melanoma patients. Serum proteomics could reveal which factors in serum of melanoma patients drive this differentiation and targeting of such factors could inhibit the differentiation of monocytes towards BDCA1+CD14+ cells [40,41]. 

As BDCA1+CD14+ cells express the coinhibitory molecule PD-L1, BDCA1+CD14+ cells could be targeted in vivo while using anti-PD-L1-coated biodegradable polymers. These targeted nanoparticles can deliver drugs to reduce their suppressive function and prevent migration into the tumor microenvironment. Anti-PD-L1 antibodies could also directly act on BDCA1+CD14+ cells. Multiple anti-PD-L1 antibodies have been approved by the FDA for clinical use and anti-PD-L1 therapy has shown effective clinical responses in cancer patients (reviewed in [42]). In melanoma, anti-PD-L1 antibodies not only act on tumor cells, but also on BDCA1+CD14+ cells. Combining DC vaccination with anti-PD-L1 treatment may be a promising strategy to further improve the efficacy of anti-cancer immunotherapy. 

The presence of BDCA1+CD14+ cells was already demonstrated in melanoma and ovarian cancer, and we now showed that they are also present in liquid or hematologic cancers such as AML and CML. We hypothesize that BDCA1+CD14+ immunosuppressive cells are of significance in a broad spectrum of cancers and therefore, it is of interest to investigate whether the BDCA1+CD14+ cells found in other cancers share the same immunosuppressive mechanisms, such as the expression of PD-L1. Future research focusing on the differentiation and biology of BDCA1+CD14+ cells could reveal new treatment opportunities and/or improvements for DC-based cancer immunotherapy. 

## Figures and Tables

**Figure A2:**
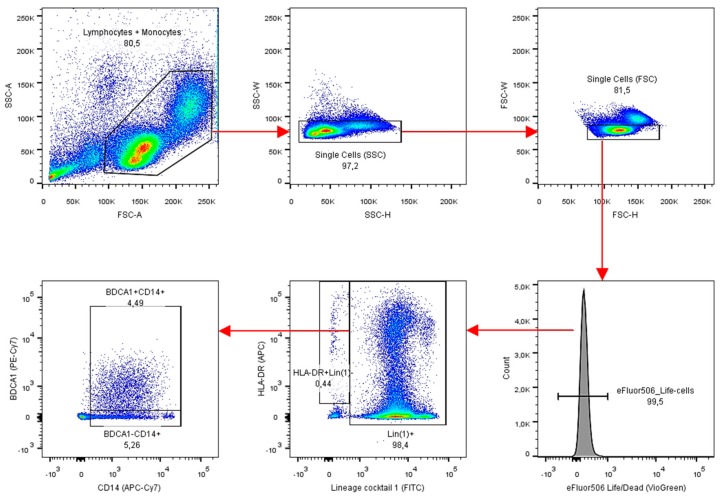
Flow cytometry gating strategy for BDCA1+CD14+ cells in PBMCs of patients with leukemia. Lymphocytes and monocytes were gated based on forward scatter and side scatter; single cells were gated on the basis of side scatter width and side scatter height followed by forward scatter width and forward scatter height; viable cells were eFluor506 negative; BDCA1+CD14+ cells were identified within the population of Lineage-positive and HLA-DR+ cells.

## Appendix A: Materials & Methods

Analysis of BDCA1+CD14+ cells in PBMCs of leukemic cancer patients was performed using flow cytometry. PBMCs were previously isolated by Ficoll density centrifugation and stored in liquid nitrogen. Upon thawing, cells were washed with X-Vivo (Lonza) and PBS. Up to 3 million PBMCs were stained with dead cell marker eFluor506-VioGreen (eBioscience). Fc-receptors were blocked with 2% human serum. PBMCs were labeled with BDCA1-PE-Cy7 (BioLegend), CD14-APC-Cy7 (BioLegend), HLA-DR-APC (BioLegend), Lineage cocktail 1-FITC (BD Biosciences) and PD-L1-PE (BD Biosciences) and measured using the FACSAria II (BD Biosciences) with BD FACSDiva Software. Spectral overlap compensation was set using positive and negative beads (MACS Comp Bead Kit; anti-mouse Igκ) on the apparatus itself. Obtained data was analyzed using FlowJo version 10. An example of the gating strategy is given in Figure A2.
